# Number Needed to Treat in Trials of Targeted Therapies for Advanced Ovarian Cancer

**DOI:** 10.1001/jamanetworkopen.2022.45077

**Published:** 2022-12-02

**Authors:** Michele Bartoletti, Sandro Pignata, Domenica Lorusso, Francesco Perrone, Diego Zara, Fabio Puglisi

**Affiliations:** 1 Unit of Medical Oncology and Cancer Prevention, Department of Medical Oncology, Centro di Riferimento Oncologico di Aviano, Istituto di Ricovero e Cura a Carattere Scientifico (IRCCS), Aviano, Italy; 2 Department of Urology and Gynecology, Istituto Nazionale Tumori IRCCS Fondazione G. Pascale, Naples, Italy; 3 Department of Women and Child Health, Division of Gynecologic Oncology, Fondazione Policlinico Universitario A. Gemelli IRCCS, Rome, Italy; 4 Department of Life Science and Public Health, Catholic University of Sacred Heart Largo Agostino Gemelli, Rome, Italy.; 5 Clinical Trials Unit, Istituto Nazionale Tumori IRCCS, Fondazione G. Pascale, Naples, Italy; 6 Department of Medicine, University of Udine, Udine, Italy

## Abstract

This comparative effectiveness study assesses the numbers needed to treat in targeted trials of agents for newly diagnosed ovarian cancer.

## Introduction

The number needed to treat (NTT), defined as the number of patients who would need to be treated to prevent an additional outcome event (eg, disease progression or death), describes absolute benefit in randomized clinical trials. The NNTs were not reported in recent studies on newly diagnosed ovarian cancer in which targeted agents, including bevacizumab and poly(adenosine diphosphate)–ribose polymerase inhibitors (PARPi), have reshaped the treatment algorithm. In this way, NNT can be useful in understanding the benefit of maintenance therapies according to biomarkers’ status.

## Methods

In this comparative effectiveness study, the NNT for progression-free survival (PFS) was calculated inverting the risk difference between survival probabilities at 12 months in the experimental and control groups of 5 pivotal trials published from 2011 to 2022 (ICON7,^1^ SOLO1,^2^ PRIMA,^3^ PAOLA-1 [Platine, Avastin and Olaparib in 1st Line],^4^ and ATHENA MONO^5^). The survival probabilities were estimated using the Kaplan-Meier survival curves with Engauge Digitizer software, version 12.1 (Mark Mitchell). The assessment of NNTs was performed in all participants and according to the homologous recombination status (deficient [HRD] or proficient [HRP]) and *BRCA* gene status, when data were available. The NNTs were rounded up to the nearest whole number. Lower NNT values indicate greater benefit. The 95% CIs for NNT were calculated as a measure of uncertainty when possible.

This study followed the International Society for Pharmacoeconomics and Outcomes Research (ISPOR) reporting guideline. The study did not require institutional review board or ethical committee review in accordance with the Common Rule because it did not involve human participants or individual patient data.

## Results

In ICON7,^1^ which tested the addition of bevacizumab to a platinum-based combination treatment against placebo, the NNT in all participants was 7 (95% CI, 5-10), whereas a nonproportional hazard ratio (HR) of 0.81 (95% CI, 0.70-0.94) was reported at the primary analysis (19.4 months follow-up). In SOLO1,^2^ olaparib was tested against placebo in *BRCA1/2*-mutated ovarian cancer. The NNT was 3 (95% CI, 2-4) at both the first (40.7 months) and second (4.8 years) analyses, with an HR of 0.30 (95% CI, 0.23-0.41). In PRIMA,^3^ niraparib was tested against placebo, and the NNTs were 6 (95% CI, 4-11) in all participants and 3 in those with *BRCA1/2* mutations. In PAOLA-1,^4^ olaparib plus bevacizumab was tested against bevacizumab alone, and the NNTs were 8 (95% CI, 5-19) in all participants and 6 (95% CI, 3-13) in patients with *BRCA1/2* mutations. In ATHENA MONO,^5^ rucaparib was tested against placebo, and the NNTs were 5 (95% CI, 3-12) in all participants and 3 (95% CI, 2-14) in patients with *BRCA1/2* mutations. Additionally, in PRIMA^3^ and ATHENA MONO,^5^ with statistically significant PFS in patients with HRP status, the NNTs were 8 with an HR of 0.68 (95% CI, 0.49-0.94) for niraparib and 7 (95% CI, 3-52) with an HR of 0.65 (95% CI, 0.45-0.95) for rucaparib (Table).

**Table.** Number Needed to Treat for PFS in Pivotal Trials of Newly Diagnosed Advanced Ovarian Cancer According to Biomarkers

Trial (source)	Experimental vs control treatments	NNT (95% CI)/survival probability at 12 mo, experimental vs control group, %				Follow-up, median (IQR)
		All participants	Participants with *BRCA1/2* mutation^a^	HRD status	HRP status	
ICON 7 (Perren et al,^1^ 2011)	Bevacizumab vs placebo	7 (5-10)/80 vs 65	NA	NA	NA	19.4 mo
SOLO1 (Banerjee et al,^2^ 2021)	Olaparib vs placebo	NA	3 (2-4)/88 vs 51	NA	NA	40.7 (34.9-42.9) mo; 4.8 (2.8-5.3) y
PRIMA (González-Martín et al,^3^ 2019)	Niraparib vs placebo	6 (4-11)/53 vs 35	3 (NA)/75 vs 44	3 (NA)/66 vs 37	8 (NA)/33 vs 20	13.8 (1-28) mo
PAOLA-1 (Ray-Coquard et al,^4^ 2019)	Olaparib plus bevacizumab vs bevacizumab	8 (5-19)/78 vs 66	6 (3-13)/93 vs 75	7 (3-204)/83 vs 68	No PFS benefit demonstrated	22.9 (18.0-27.7) mo
ATHENA MONO (Monk et al,^5^ 2022)	Rucaparib vs placebo	5 (3-12)/61 vs 42	3 (2-14)/81 vs 52	5 (2-58)/65 vs 44	7 (3-52)/50 vs 36	26.1 (24-27.7) mo

Abbreviations: HRD, homologous recombination deficient; HRP, homologous recombination proficient; NA, not available; NNT, number needed to treat; PFS, progression-free survival.

^a^ Excluding germline and somatic *BRCA* mutations.

## Discussion

These findings suggest that the magnitude of benefit from targeted therapies in newly diagnosed ovarian cancer is deeply affected by the biomarkers’ status. Patients with *BRCA* mutations benefit most from PARPi, and adding bevacizumab seems to provide no further advantages. A PARPi-based targeted therapy is also recommended in patients with HRD status, given the clear benefit in avoiding disease progression. In this case, the choice of adding bevacizumab to olaparib vs niraparib or rucaparib alone should also consider potential adverse effects, contraindications, local reimbursement policy, and postprogression strategy. For patients with HRP status, a growing body of evidence supports the use of PARPi, and other mechanisms of action of PARPi beyond synthetic lethality should be unveiled. However, cross-trial comparisons should be conducted with caution because the characteristics of patients included in these trials and follow-up length were different. Finally, despite the actual treatment algorithm largely based on PFS data, PFS has not been established as a surrogate end point for overall survival (at least at a trial level and before the introduction of targeted agents),^6^ and in the recurrent setting, niraparib as maintenance and rucaparib as active therapies have failed to demonstrate survival benefits. Future research should discover disease vulnerability in the HRP subgroup.

## Notes

Supplement.
Data Sharing Statement
