# Supplementary material for: Number Needed to Treat in Trials of Targeted Therapies for Advanced Ovarian Cancer
Source: JAMA Netw Open. 2022 Dec 2;5(12):e2245077. doi: 10.1001/jamanetworkopen.2022.45077 (PMC9719047; doi:10.1001/jamanetworkopen.2022.45077)
Supplement: Supplement. — Data Sharing Statement [file jamanetwopen-e2245077-s001.pdf]

Data Sharing Statement

Bartoletti. Number Needed to Treat in Trials of Targeted Therapies for Advanced Ovarian Cancer. *JAMA Netw Open*. Published December 2, 2022. doi:10.1001/jamanetworkopen.2022.45077

Data
Data available: No

Additional Information
Explanation for why data not available: All data used in the estimation of NNT are reported in the article. No additional data have been used.
